# Deconvolution of the density of states of tip and sample through constant-current tunneling spectroscopy

**DOI:** 10.3762/bjnano.2.64

**Published:** 2011-09-19

**Authors:** Holger Pfeifer, Berndt Koslowski, Paul Ziemann

**Affiliations:** 1 Institut für Festkörperphysik, Universität Ulm, D-89069 Ulm, Germany

**Keywords:** deconvolution, Nb DOS, STM, STS

## Abstract

We introduce a scheme to obtain the deconvolved density of states (DOS) of the tip and sample, from scanning tunneling spectra determined in the constant-current mode (*z*–*V* spectroscopy). The scheme is based on the validity of the Wentzel–Kramers–Brillouin (WKB) approximation and the trapezoidal approximation of the electron potential within the tunneling barrier. In a numerical treatment of *z*–*V* spectroscopy, we first analyze how the position and amplitude of characteristic DOS features change depending on parameters such as the energy position, width, barrier height, and the tip–sample separation. Then it is shown that the deconvolution scheme is capable of recovering the original DOS of tip and sample with an accuracy of better than 97% within the one-dimensional WKB approximation. Application of the deconvolution scheme to experimental data obtained on Nb(110) reveals a convergent behavior, providing separately the DOS of both sample and tip. In detail, however, there are systematic quantitative deviations between the DOS results based on *z*–*V* data and those based on *I*–*V* data. This points to an inconsistency between the assumed and the actual transmission probability function. Indeed, the experimentally determined differential barrier height still clearly deviates from that derived from the deconvolved DOS. Thus, the present progress in developing a reliable deconvolution scheme shifts the focus towards how to access the actual transmission probability function.

## Introduction

Undoubtedly, the power of scanning tunneling microscopy (STM) is based on its capability to map the surface topology of a conductive sample with resolution down to the atomic scale in real space [1]. Moreover, previous experience on metal–insulator–metal tunnel junctions [2] immediately suggested extending STM to become a local analytical tool, opening up the field of scanning tunneling spectroscopy (STS). The most prominent property that can be accessed by STS is the local electronic density of states (LDOS). For that purpose, the applied tunneling bias, *V*, is ramped while the probe–sample separation is kept constant (commonly denoted as *I*–*V* spectroscopy) [1,3]. However, determination of the sample LDOS from such a measurement is always obscured by unavoidable interfering influences from other STS constituents such as the tunneling barrier, with its bias-dependent transmission probability, as well as from the LDOS of the probing tip. The problem becomes most clearly visible when referring to the semiclassical Wentzel–Kramers–Brillouin (WKB) description of tunneling processes. There, the experimentally determined tunneling current, *I*, is expressed as a convolution integral involving the sample and tip LDOS as well as the barrier behavior on equal footing. Thus, if there is just one *I*–*V* characteristic available for a given tunneling barrier, extraction of the sample LDOS is in principle impossible. That used to be the standard situation for previous tunnel junctions with their fixed oxide barriers. In STS, however, at any given sample location, barriers can be experimentally adjusted. In this way, additional information is provided by taking *I*–*V* curves at different fixed *z* values.

Based on this additional degree of freedom when applying STS, much work has been devoted in the past to unraveling the different contributions to the tunneling current. Most of the work was concerned with removing at least a proposed tunneling probability from the measured quantity, assuming validity of the WKB approximation. This, however, still leaves a kind of a convolved LDOS of tip and sample [4–12]. Recently, it was shown that the tunneling current as described by the WKB approximation can be transformed into a Volterra integral equation of the second kind and, therefore, well-known schemes can be applied to solve such an equation numerically [7–8]. Taking this one step further, it was demonstrated that, taking *I*–*V* curves at different tip–sample distances, these Volterra equations form a set of coupled integro-differential equations, which allow for a deconvolution of the transmission probability as well as of the LDOS of tip and sample [13].

*I*–*V* spectroscopy is not the only STS measurement mode to determine the LDOS of a sample. Though less commonly used, *z*–*V* spectroscopy, alias constant-current spectroscopy, offers an interesting alternative to the *I*–*V* mode. In constant-current mode, the topographic feedback loop is left on and, thus, the tunneling current is held constant while the bias, *V*, is scanned and the derivative of *I* with respect to *V*, ∂*_V_I*(*V*), as well as the varying tip–sample separation, *z*(*V*), is acquired. In a number of cases, *z*–*V* may be superior to *I*–*V* spectroscopy. Examples are the common case of a limited dynamic range in the current measurement, the need to keep the tunneling current below a certain upper limit in order to avoid local damage of a delicate sample, or the necessity of a measurement over a wide range of biases, such as in case of samples with adsorbed organic molecules in order to resolve the lowest unoccupied molecular orbital (LUMO) [14–16]. Ziegler et al. showed that an approximation, originally derived for *I*–*V* spectroscopy in reference [7], can be used to obtain a joint LDOS of tip and sample from such a measurement [17]. However, so far such a deconvolution scheme as that for *I*–*V* spectroscopy is not yet available for *z*–*V* spectroscopy.

Consequently, it is the aim of the present contribution to provide such a scheme by extending the previous deconvolution derived for *I*–*V* spectroscopy and tailoring it for the *z*–*V* mode*.* After a short introduction to the theory, the deconvolution scheme is applied to different LDOS model functions for tip and sample. By numerically calculating the related *I*–*V* curves for various barrier and LDOS parameters, insight is gained into how these parameters may influence “experimental” data. The newly developed deconvolution is adopted to analyze experimental STS data obtained on Nb(110). By comparison with earlier results we find that the commonly used transmission probability function (TPF) according to the one-dimensional WKB approximation is deficient, at least in the case of Nb(110). We propose that the differential barrier height is a sensitive indicator of how to adjust the TPF to obtain a more realistic description of the tunneling probability.

## Results and Discussion

### Theory

The underlying theory for *z*–*V* spectroscopy is closely related to that applied to *I*–*V* spectroscopy [7] except for an explicit dependence on the tip–sample separation, *z*. The starting point of the calculation is the tunneling current, *I*, as given by the one-dimensional WKB approximation for a barrier characterized by an energy-dependent transmission probability function (TPF), *T*(*E*,*V*,*z*). Assuming zero temperature, application of a bias, *V*, results in a tunneling current which, according to WKB, can be written as

[1]
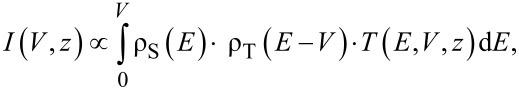


where ρ_S_ and ρ_T_ are the sample and tip density of states (DOS), respectively, and *E* is the energy of electrons participating in the tunneling process; *z* is considered as being an independent variable with ∂*_V_z* = 0. If, in a *z*–*V* measurement, *z* is the response of the tip–sample separation on ramping the bias voltage for a preset constant current, *I*_0_, we denote *z* as 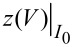 or for simplicity *z*(*V*). According to Simmons [18–19] the TPF, *T*(*E*,*V*,*z*), can be approximated by assuming a trapezoidal shape of the barrier, leading to

[2]
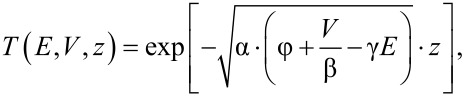


where φ is the height of the tunneling barrier at zero bias and 
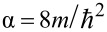
. We introduced here the dimensionless parameters β and γ. For the trapezoidal approximation in 1-D, β = 2 and γ = 1. The parameter β may be increased slightly, e.g., to account for the image potential, while γ may be used to consider the energy dependence of the TPF on the dispersion relation of the electrons [7,20]; for a surface state with no energy component perpendicular to the surface and perfect parabolic dispersion, γ = 0. Note, that γ × *E* may be replaced by any continuous energy-dependent function with no impact on the proposed formalism. This way, a wide range of energy-dependent decay lengths of the electron states into the vacuum may be implemented in the deconvolution scheme.

Taking the derivative of Equation 1 with respect to bias delivers

[3]
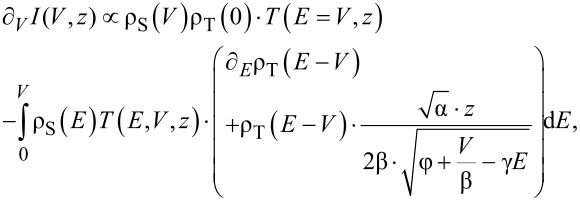


where we used −∂*_V_*ρ(*E*−*V*) = ∂*_E_*ρ(*E*−*V*). Comparing Equation 3 with Equation 4 in [17] we set ∂*_V_z* = 0 since *z* is an independent variable. If we set *z* = 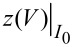, ∂*_V_I*(*V*,*z*(*V*)) would necessarily be zero. Equation 3 can now be solved formally for ρ_S_(*V*), giving

[5]
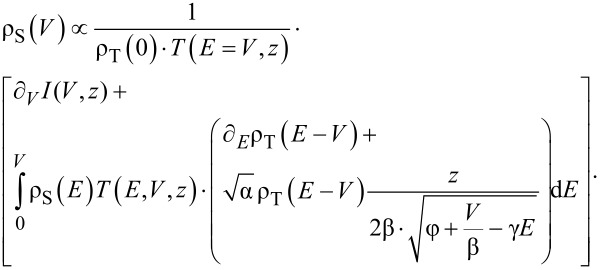


This is a Volterra integral equation of the second kind. For a known ρ_T_(*V*) it can be solved numerically by means of the Neumann approximation scheme, by replacing ρ_S_(*V*) on the left side by ρ_S_(*V*)*_n_*_+1_ and on the right side by ρ_S_(*V*)*_n_*. Assuming a constant tip DOS, φ > *V*, and applying the mean value theorem of integrals, we arrive at an equation that has been introduced already in [7] for *I–V* spectroscopy and extended to *z–V* spectroscopy in [17]:

[4]
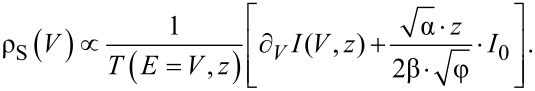


As the tunneling junction is symmetric, we may change the reference frame from the sample to the tip. We then obtain similarly a Volterra integral equation for the tip DOS:

[6]
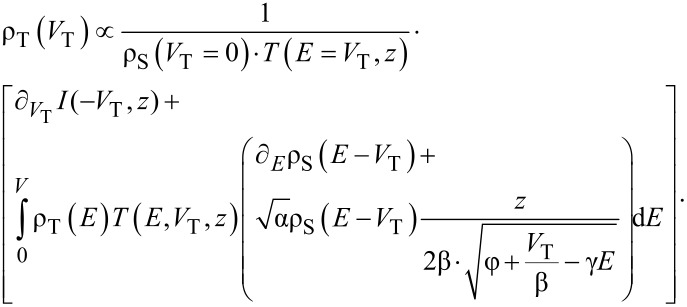


where *V*_T_ = −*V* is the bias with respect to the Fermi level of the tip. Note that this equation is identical to Equation 5 in the respective reference frame, but ∂*_V_I* has to be mirrored at *V* = 0 because ∂*_V_I* has been measured in the reference frame of the sample.

With Equation 5 and Equation 6 we have a set of two coupled integro-differential equations for the sought properties ρ_S_(*V*) and ρ_T_(*V*) based on the measurable data ∂*_V_I* and *z*(*V*). The additionally required parameter φ can be determined from, e.g., *I–z* spectroscopy and the absolute tip–sample separation, 
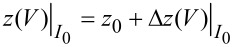
, can be reasonably guessed or estimated through recently proposed methods [12].

This set of Volterra integral equations is applied in order to deconvolve the DOS of both sample and tip, by referring to a previously described scheme [13]. There, the basic idea was to take advantage of the additional information provided by different ∂*_V_I*–*V* curves, taken at different tip–sample separations. Presently, the additional information is provided by different ∂*_V_I*–*V* and *z*(*V*) curves taken at different set currents, *I*_0_. Note that it is the TPF that makes the difference. As it is not symmetric in *E*, *V*, or *z*, it allows deconvolution of the tip and sample DOS .

In the following section we will demonstrate this deconvolution scheme for *z*–*V* spectroscopy. Starting with model functions for ρ_S_(*V*) and ρ_T_(*V*), the “experimental” data *I*(*V*,*z*) for a given *V* and *z* are calculated according to Equation 1. Additionally, 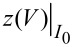 for a given set current, *I*_0_, is determined by calculating *I*(*V*,*z*) and varying *z* until |*I*(*V*,*z*)–*I*_0_|/*I*_0_ < 0.001. Next, ∂*_V_I* is calculated from Equation 3 with the afore-determined 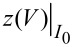 for a given *I*_0_. In this way, two sets of ∂*_V_I* and *z*(*V*) curves for set currents *I*_0,1_ and *I*_0,2_ are obtained and mimic experimental data, which form the starting point of the deconvolution procedure. Assuming that on entry to the deconvolution scheme no specific information is available for ρ_S_(*V*) and ρ_T_(*V*), both entries are initialized as unity for the iteration.

It should be noted that, due to the coupled equations, one may commence the iteration with data for either the larger or the smaller set current. There is no criterion available to determine a better choice. In the following examples we achieved better results, i.e., faster convergence and better accuracy, when starting with data for the smaller set current. Note also, as previously reported [7,13], that numerical errors may accumulate during the iterations leading to a divergence of the DOS at the boundaries. For a sufficient optimization we found that it is necessary to repeat the iteration at least four times but not more than six times, which, according to the numerical examples presented below, will lead to acceptably accurate results.

Equation 6 and Equation 5 exhibit an apparent singularity with *z* → −∞ for *V* → 0. However, this singularity is not substantial in theory and is of no practical relevance, since experimental data can be safely measured only above a minimal bias, *V*_min_. Below *V*_min_ the tip or sample could be damaged. Assuming that the measurement is started at that sufficiently small bias, *V*_min_, such that ρ_S_, ρ_T_ ≈ constant for |*E*| < |*V*_min_| and *V*_min_ << φ, we replace in Equation 5 the integral


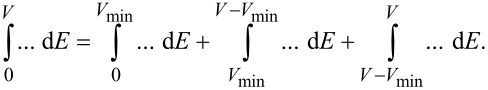


The first term on the right then gives


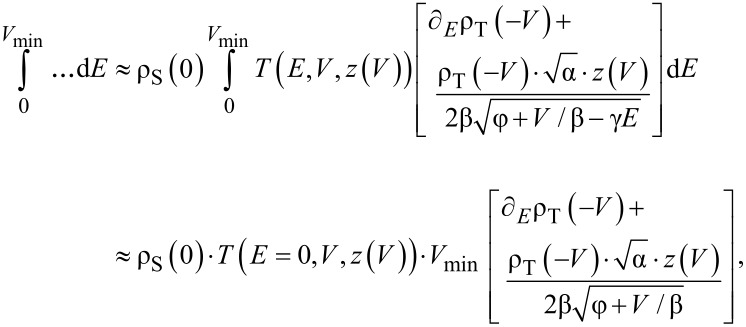


and similarly the last term gives


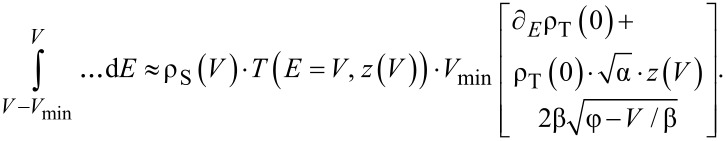


These approximations simplify the numerical integration of experimental data. However, to account for the missing data in the range 0 ≤ *V* ≤ *V*_min_, extrapolation of the experimental data towards zero bias is carried out. For this purpose, in the case of ∂*_V_I* being linear and for *z*(*V*) being logarithmic the extrapolations delivered satisfying results.

### Numerical Examples

#### Analysis of peak positions

**Figure 1:**
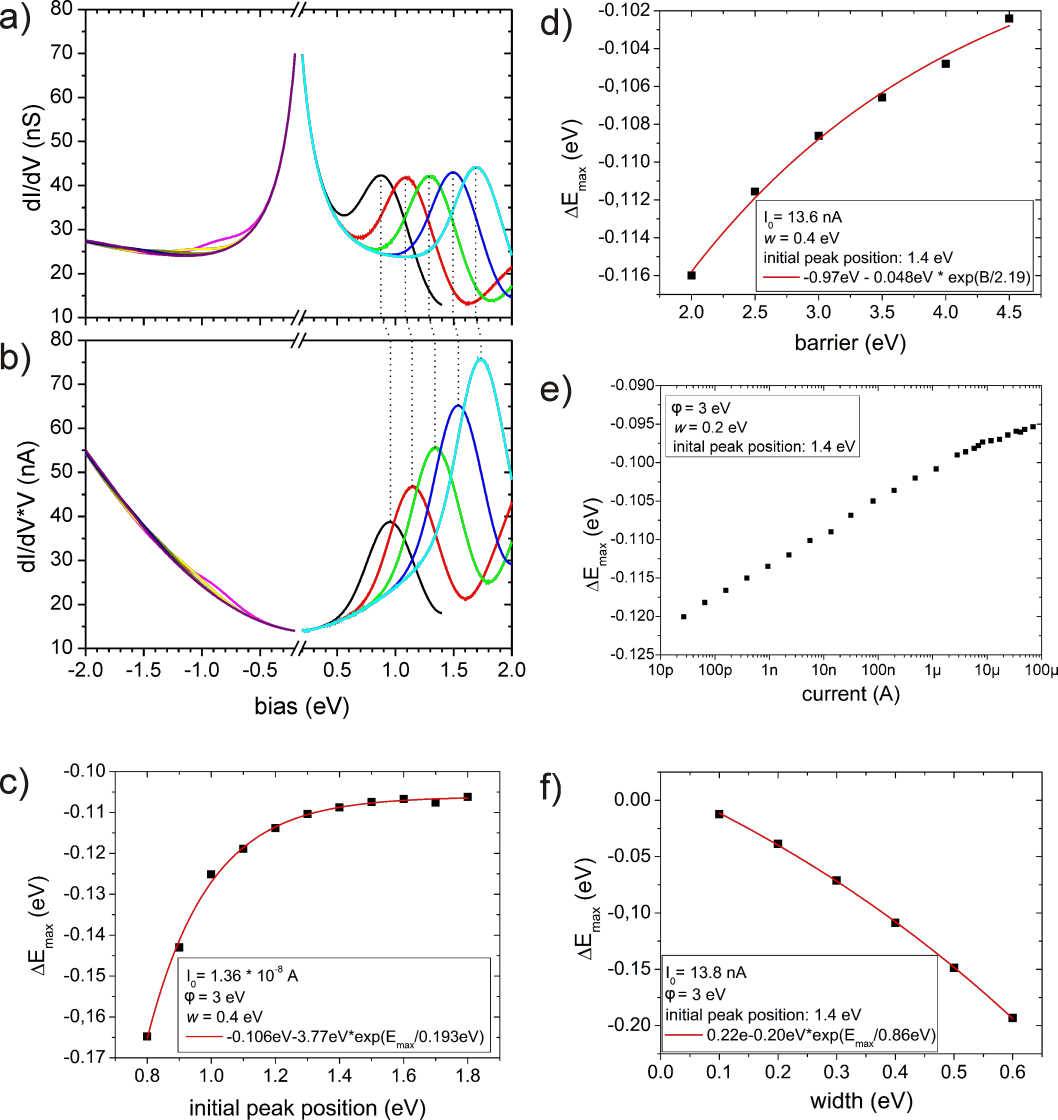
Calculated ∂*_V_I–V* curves (a) and (∂*_V_I* × *V*)*–V* curves (b) for different peak positions in the sample DOS ranging from −1.8 eV to +1.8 eV. The model DOS of tip and sample are constant with an additional Gaussian peak in the sample DOS at the given position. Parameters are: Barrier height φ = 3 eV, *I*_0_ = 13.7 nA. The initial peak positions were ±[1.0 (black, magenta), 1.2 (red, yellow), 1.4 (green, dark yellow), 1.6 (blue, navy), 1.8 (cyan, purple)] eV. Panels (c) through (f) show the dependence of the peak positions in ∂*_V_I* on the barrier height, φ, the set current, *I*_0_, the initial peak position, *E*_max,0_, and the width of the peak, *w*, respectively. The values of the set currents result from the particular choice of the tip–sample separation at zero bias.

Before we start applying the deconvolution procedure, we analyze how different characteristic features of the sample and tip DOS appear in the “experimental” ∂*_V_I*–*V* curves or the derived quantity ∂*_V_I* × *V* within the one-dimenional WKB approximation. For that purpose, we start with a Gaussian peak of a given width and height, at various peak positions within the range −1.8 eV to +1.8 eV, in an otherwise constant DOS of the sample while the tip DOS is kept constant. In Figure 1a the corresponding conductivity, ∂*_V_I*, is presented showing the following characteristics: (i) There is a hyperbolic background in the conductivity, since the TPF can be approximately described by *T* ~ *V*^−1^ at low bias for a constant DOS and constant tunneling current (the already discussed singularity at zero bias is excluded). (ii) For DOS peaks in the negative energy range (occupied states) only weak shoulders are visible, while DOS peaks in the positive energy range (empty states) result in pronounced peaks with increasing amplitude for increasing peak bias positions. This is an immediate consequence of the growing TPF for increasing energies, *E*. In the present case, the resulting conductivity peaks in the negative bias range are so weak that a reasonable analysis of their position is not possible. (iii) The pronounced peaks in ∂*_V_I* at positive bias shift in energy with respect to the original position in the DOS. The shift is always negative and amounts to −0.15 eV in Figure 1a. Furthermore, this peak shift depends on the position, *E*_max,0_ of the Gaussian in the model DOS as well as on its width, *w*, the set current, *I*_0_, and the barrier height, φ. The corresponding numerically determined peak positions, as depending on these parameters, are presented in Figure 1c–f. Due to the hyperbolic background, the peak shift in ∂*_V_I* is larger the closer the original DOS peak is to zero bias. The dependence on its width, *w*, and the set current, *I*_0_, is as expected: The broader the original DOS peak and the smaller the set current, the larger the shift of the corresponding peak in ∂*_V_I*. Thus, it is worth noting that for lifetime-broadened states in transition metals or molecular states on top of a metal surface, which may approach ~1 eV, the expected peak shifts in ∂*_V_I* will be of similar order. The peak shift in dependence on the barrier height (Figure 1d) is larger the lower the barrier. However, for a reasonable barrier height ranging from 2 eV to 4.5 eV the shift is relatively small.

The hyperbolic background around zero bias can be removed by plotting ∂*_V_I* × *V* versus *V* (Figure 1b), which corresponds to setting *I*_0_ = 0 and *T ~ V*^−1^ in Equation 4. Accordingly, the peaks at positive bias shift back towards the original positions as given by the sample DOS. Peaks in the negative bias range, however, remain hardly detectable while those in the positive range are largely enhanced at increasing bias positions, even though the peak heights are all equal in the original DOS. Thus, the results presented in Figure 1 may serve as a warning that experimentally observed peak-like features in ∂*_V_I* cannot immediately be assigned to corresponding DOS characteristics. For that purpose, a complete deconvolution procedure must be applied as presented in the following.

#### Recovery and deconvolution of the DOS

**Figure 2:**
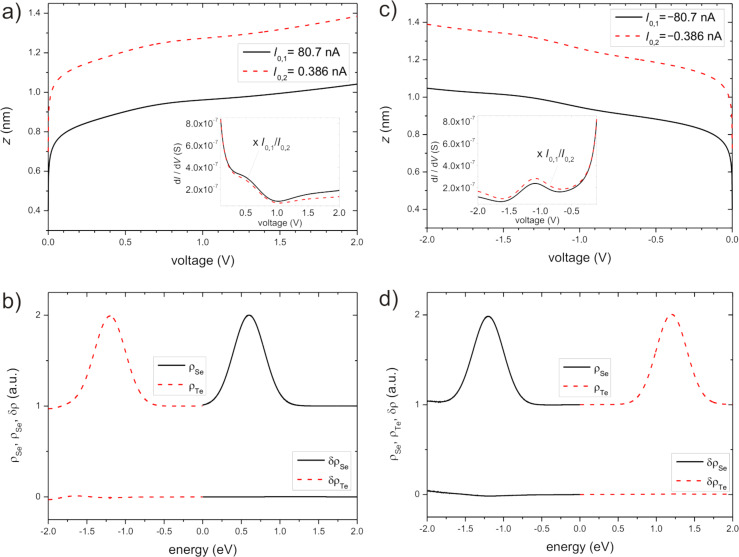
Numerically calculated *z*(*V*)–*V* curves in the a) positive and c) negative bias range with respect to the sample. The two set currents are a) *I*_0,1_ = 80.7 nA and *I*_0,2_ = 0.386 nA and c) *I*_0,1_ = −80.7 nA and *I*_0,2_ = −0.386 nA. The inset displays the corresponding ∂*_V_I*–*V* curves. For better comparison, the ∂*_V_I*–*V* curve for *I*_0,2_ has been scaled by *I*_0,1_/*I*_0,2_. b) and d) are the corresponding recovered and deconvolved DOS for the positive and negative bias range, respectively, together with the errors (δ) relative to the model DOS (upper curves, ρ_Se_: black solid, ρ_Te_: red dashed; lower curves, δρ_Se_: black solid, δρ_Te_: red dashed). The values of the set currents result from the particular choice of the tip–sample separation at zero bias.

In the first example we focus on the positive bias range (in the reference frame of the sample). Again, a model DOS of the sample is defined by setting it to unity with an additional Gaussian peak centered at 0.6 eV (height *h* = 1, width *w* = 0.2 eV). Similarly, the model DOS of the tip is taken as unity with a Gaussian peak at −1.2 eV (*h* = 1, *w* = 0.2 eV). For two set currents *I*_0,1_ = 80.7 nA and *I*_0,2_ = 0.386 nA, and an effective barrier height of φ = 3 eV the resulting *z*–*V* curves and the corresponding ∂*_V_I–V* curves were calculated. The results are displayed in Figure 2a and represent “experimental” data as before. The curves show the expected behavior: Decreasing the set current from *I*_0,1_ to *I*_0,2_ leads to a shift of *z*(*V*) to larger values. The ∂*_V_I–V* curves (inset in Figure 2a) roughly scale with the ratio of the set currents. In detail, however, there are small deviations from a constant shift of *z*(*V*) or from a linear scaling of ∂*_V_I* with the set currents. It is exactly these deviations that allow for the deconvolution of the DOS. Both *z*–*V* curves exhibit a logarithmic behavior with two shoulders, one at about 0.7 eV and a very small one at 1.6 eV. The conductivity ∂*_V_I* shows a shoulder at around 0.5 eV and a broad peak at around 1.5 eV, both on top of a hyperbolic background (~ *V*^−1^) as discussed above.

When applying the deconvolution scheme to these data it turned out that the best results were achieved by starting the iteration from Equation 5 with data related to the smaller set current, *I*_0,2_. After six iterations the Gaussian peaks in the sample and tip DOS clearly emerged (Figure 2b) with an accuracy of better than ±0.03 when compared to the given model DOS.

As a second example, the deconvolution scheme was applied to the negative bias range (in the reference frame of the sample). Again, the model DOS used as input consists of a Gaussian peak on top of a constant background at unity. The peak in the sample DOS is centered at −1.2 eV (*h* = 1, *w* = 0.2 eV) and in the tip DOS at +1.2 eV (*h* = 1, *w* = 0.2 eV). It should be noted, that both peaks should lead to a superposed feature appearing at a bias of −1.2 eV in the “experimental” ∂*_V_I* curves. With the two set currents, *I*_0,1_ = −80.7 nA and *I*_0,2_ = −0.386 nA, we obtained the two *z*–*V* curves displayed in Figure 2c. Both *z*–*V* curves show a logarithmic behavior with a shoulder at about −1.2 eV. The related ∂*_V_I*–*V* curves are displayed in the inset of Figure 2c. Both have a peak at −1.1 eV. The shift of about 0.1 eV relative to the model value has already been discussed in section “Analysis of peak positions”. When applying our deconvolution scheme at negative bias the best results were achieved when starting from Equation 6 with data for the lower set current. After six iterations the deconvolved DOS of tip and sample emerged as shown in Figure 2d. In comparison to the model DOS the accuracy is better than 0.0042 over the complete bias range under study.

Both examples clearly demonstrate successful recovery and deconvolution of the density of states of tip and sample within the framework of the one-dimensional WKB approximation with data obtained from *z*–*V* spectroscopy. As in the case of *I*–*V* spectroscopy, we found (besides numerical/technical issues) only one restriction to the successful implementation of the formalism: The DOS of tip and sample must be continuously differentiable, and slopes that are too steep will reduce the accuracy of the result. It turned out, as might have been expected, that the larger the difference between the two set currents the better the recovery and the deconvolution of the DOS of the tip and sample.

### Application to experimental data

In the following we apply the above-described scheme to experimental data obtained on Nb(110) [21]. The measurements were performed on a home-built low-temperature STM operated at a base pressure of ~10^−10^ mbar and a base temperature of 5.2 K [22]. The ∂*_V_I–V* curves were recorded at different set currents by employing a lock-in technique with a modulation frequency of ~500 Hz, which is well above the bandwidth of the topographic feedback loop. As tunneling tip, we used an electrochemically etched tungsten wire, which was subsequently heated in UHV to ~2000 °C and conditioned by field emission and desorption.

**Figure 3:**
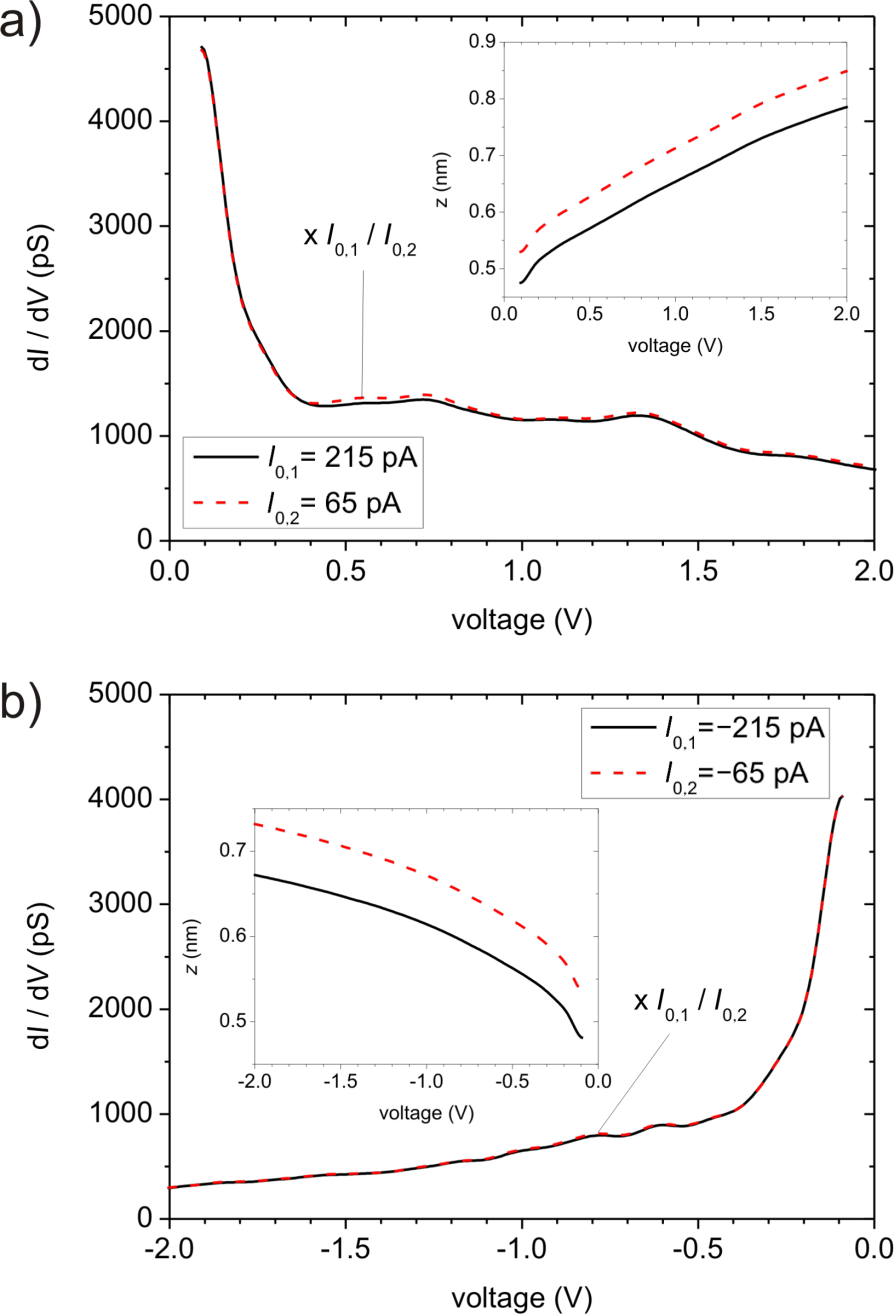
∂*_V_I-V* curves measured on Nb(110) a) in the positive bias range at two different set currents (*I*_0,1_ = 215 pA and *I*_0,2_ = 65 pA), and b) in the negative voltage range at *I*_0,1_ = −215 pA and *I*_0,2_ = −65 pA. The insets depict the corresponding *z*(*V*)–*V* curves, which were measured simultaneously. (φ = 4.1 eV). The given values of the set currents are the averages of the tunneling current during the measurement at an accuracy of ±5%.

**Figure 4:**
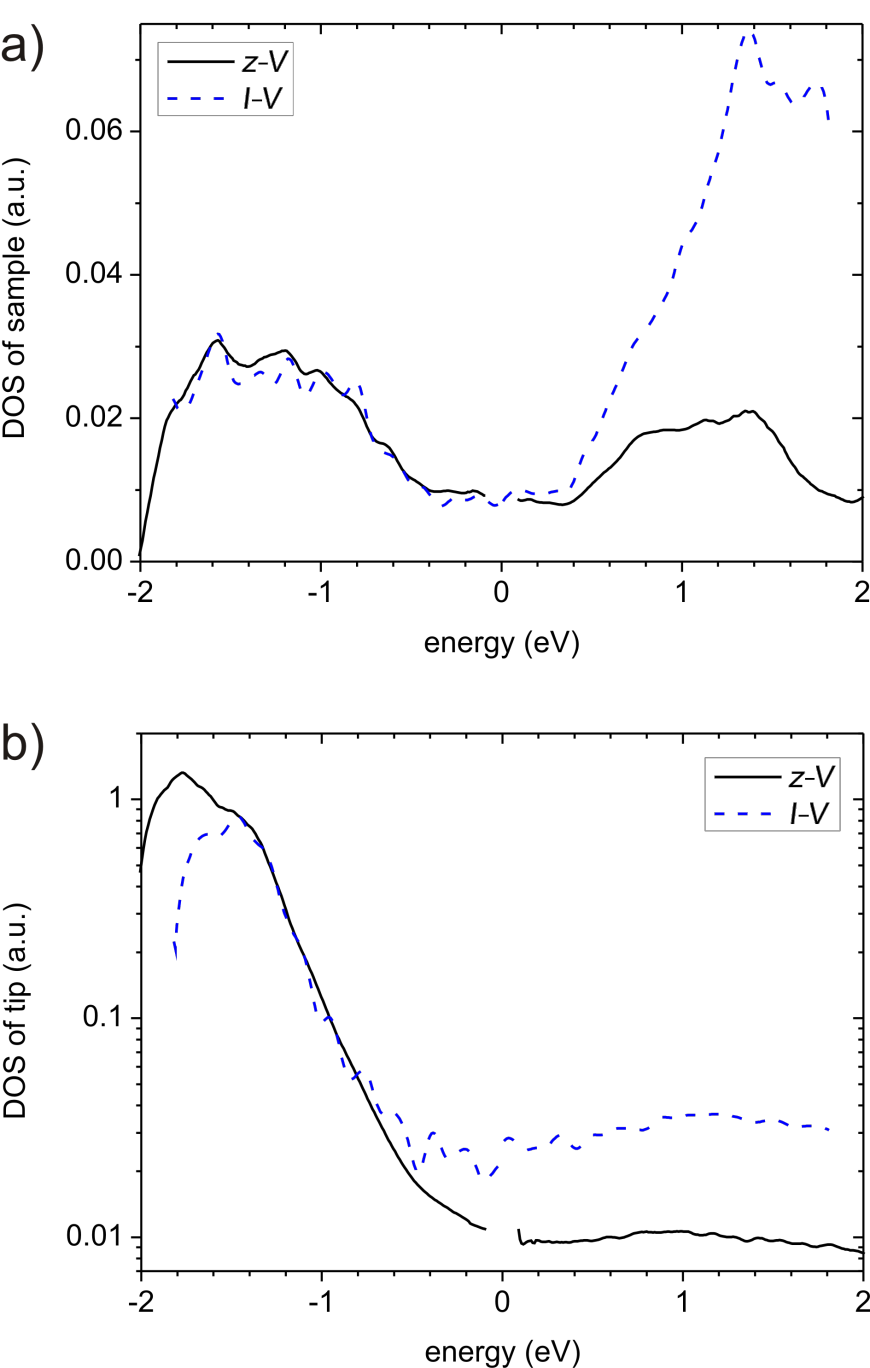
Recovered and deconvolved DOS’s of the sample derived from *z*–*V* measurements (black solid) and *I*–*V* measurements (dashed). Parameters used in the calculations are: *z*_0_ = 0.4 nm for *z*–*V* and *z*_0_ = 0.6 nm for *I*–*V* spectroscopy, β = 2, γ = 1.11 for positive and γ = 1.0 for negative energy.

In analogy to [13] we measured a set of five ∂*_V_I–V* curves together with the corresponding *z–V* curves for both positive and negative bias. For each sign of the bias, two of these ∂*_V_I–V* curves are displayed in Figure 3. The ∂*_V_I–V* curves for the smaller set current, *I*_0,2_, have been scaled by the ratio of the set currents, *I*_0,2_/*I*_0,1_, in order to make the small separation-dependent differences apparent. The insets depict the corresponding *z–V* curves, which were recorded simultaneously. We applied the deconvolution scheme pairwise to the data sets, on each occasion cycling three times through the iteration in order to avoid divergence of the DOS at the interval boundaries. The resulting tip DOS’s were averaged and the result, ρ_Te_ (cf. Figure 4b), was used to calculate the sample DOS. The averaged result for the sample is presented in Figure 4a as “experimental sample DOS”, ρ_Se_. The tunneling barrier height was determined from a separate *I–z* measurement at low bias at the same spot as the ∂*_V_I–V* curves, and a value of φ = 4.1 eV was extracted. The coefficients β and γ in the TPF (Equation 2) were set to β = 2 and γ = 1 for negative energy and γ = 1.11 for positive energy. This choice of γ for positive energy results from an adjustment of the tunneling probability to the measured differential barrier height, as proposed in [20] (see below).

Inspecting first ρ_Se_, one finds two broad maxima centered at around ±1.2 eV with a plateau in between from −0.5 eV to +0.4 eV. There are several minor peaks and shoulders in the negative energy range at [−1.8, −1.6, −1.25, −1.0, −0.8, −0.6, −0.3, −0.15] eV, and at [0.5, 0.8, 1.1, 1.4, 1.8] eV in the positive energy range. Most probably due to the limited accuracy of data and/or the calculation, the specific development of those features depends on the course of the iteration and the details of the data processing. The tip DOS, ρ_Te_ (Figure 4b), is smooth in the positive energy range, with a weak and broad maximum at about 1 eV. In the negative energy range, however, there is an exponential increase with a pronounced maximum at −1.8 eV.

For comparison we performed additionally constant-separation spectroscopy at the same location and applied the related deconvolution scheme [13] to those data using the same parameters β and γ. The result is included in Figure 4 (dashed blue lines). It agrees very well with the DOS obtained from *z*–*V* spectroscopy except for a much stronger increase (a factor of ~7 instead of a factor of 2) in the sample DOS at positive energy. We interpret this as an indication that the assumed TPF needs to be modified. The stronger increase in the sample DOS is related to the less pronounced peak of the tip DOS at negative energy. It is important to note that the energetic position of the characteristic DOS features is almost identical in *I*–*V* and *z*–*V* spectroscopy. The features, however, are palpably more pronounced in *I*–*V* spectroscopy. Comparing results obtained here with those published previously [13,21] or with theory [23–26], the energetic positions are similar, but the overall behavior of the DOS is considerably different. Consequently, in the following we focus on the overall behavior of the DOS.

There are several reasons why the experimental DOS obtained here may or even should be different from the theoretical/expected DOS. Firstly, with STM local measurements are performed revealing correspondingly local DOS variations due to the imperfections of a real Nb(110) surface. Secondly, one should be aware that at negative energies, i.e., for occupied states, recovery and deconvolution of the DOS is much more challenging, because these states contribute little to the total tunneling current. This is most clearly demonstrated in Figure 1a and b, where identical peaks in the sample DOS change from being pronounced in STS spectra when centered at positive energies, to being hardly detectable when shifted to negative energies. As a consequence, even small deviations from our assumptions (1-D WKB and trapezoidal approximation; dispersion) or measurement errors have a strong impact on the result, especially in the negative energy range (on the sample and the tip side). A rough estimate of the required accuracy in the measurement is given by considering the fractional contribution of the TPF to the total ∂*_V_I*, δ_c_ that arises from electron states at the Fermi level of the tip compared to that which arises from the states at the Fermi level of the sample. This contribution is approximately given by


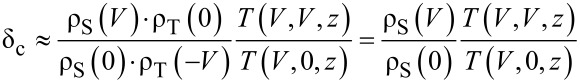


assuming ρ_T_(*E*) = 1 and *V* << 0 V. If now ρ_S_(*E*) = 1 + δ_S_Θ(*E − V*) with the Heaviside function, Θ, i.e., ρ_S_ changes at *E* = *V* by a step of size *−*δ_S_ to unity, the contribution of states at energy *E* = *V* changes at bias, *V*, by


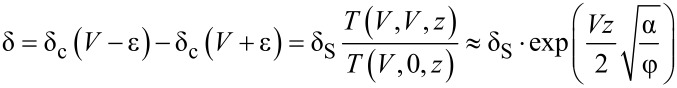


Solving for *V* and setting the inequality correctly, leads to

[7]
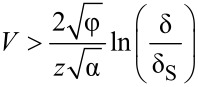


δ corresponds to the required accuracy to detect a relative change of δ_S_ in the sample DOS at bias *V*. In our experiments, an accuracy of 1% corresponds to a detectable change of ~9% in the sample DOS at −2 eV. The accuracy of the deconvolved DOS is probably even lower.

**Figure 5:**
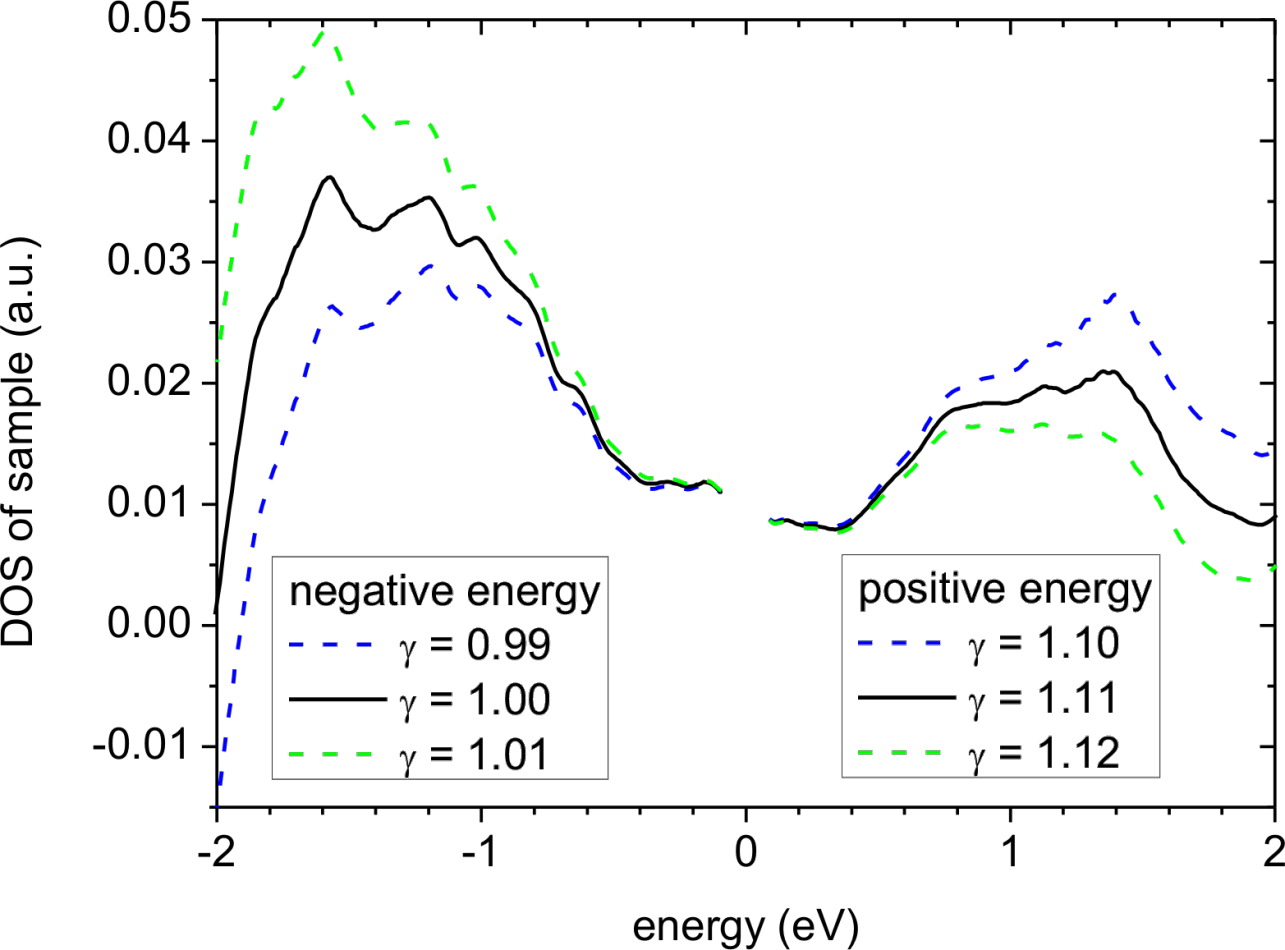
Influence of the parameter γ on the resulting experimental sample DOS. Three DOS are shown in the positive energy range for γ = 1.11 as in Figure 3a (black solid curve), γ = 1.10 (blue dashed), γ = 1.12 (green dashed) and in the negative energy range for γ = 1.00 as in Figure 3a (black solid curve), γ = 0.99 (blue dashed), γ = 1.01 (green dashed). Note that changing γ has an asymmetric influence on the DOS.

A third and probably the most important reason why the experimental sample DOS would be different from expectations is the fact that we used effectively a one-dimensional WKB approximation. To correct for this to first order, we introduced the parameter γ in Equation 2 where, for the sake of simplicity, we assumed γ to be constant, i.e., the parallel energy component *E*_p_ = (1 − γ)*E*. Such a correction has a tremendous impact on the resulting DOS especially at negative energy. In order to elucidate that problem, we show in Figure 5 a comparison of the experimental sample DOS displayed in Figure 4 (black curve) with results of the calculation obtained by changing γ just by ±0.01. Such a tiny change of γ leads to a variation of the DOS by ±40% at +2 eV, and at −2 eV the DOS even goes negative. Thus, the difficulty shifts from the original problem of developing a deconvolution scheme to the problem of finding the correct TPF.

**Figure 6:**
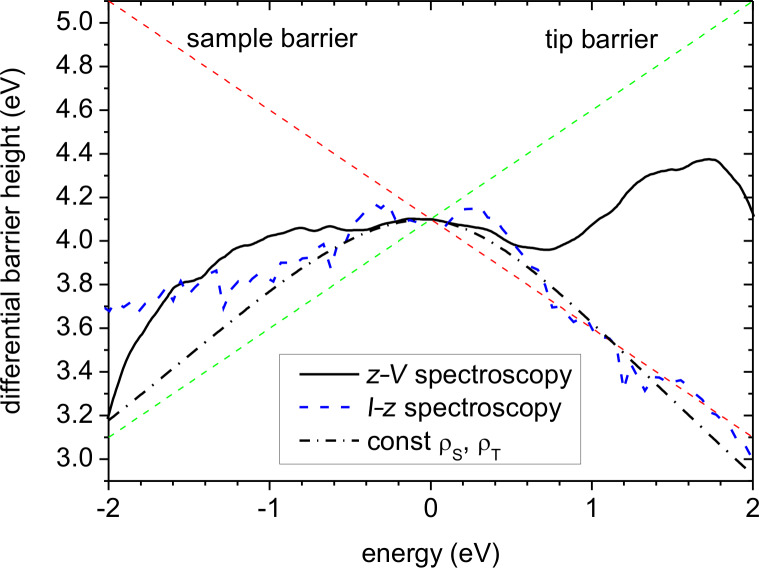
Differential barrier height (*z* = 0.6 nm, φ = 4.1 eV) as derived from the deconvolved DOS (Figure 4, solid curves) according to the WKB approximation, and as obtained from an independent ∂*_V_I*–*z* measurement with the same tip on the same sample at the same position (dashed blue). The black dash-dotted curve is the calculated DBH for constant DOS using the same TPF as for the other data, i.e., β = 2, γ = 1.11 for positive bias and γ = 1.0 for negative bias. The sample and tip barriers indicate the barriers experienced by electrons at the Fermi level of the tip and of the sample, respectively, according to the one-dimensional WKB approximation.

Elaborating on that point, we employ the differential barrier height (DBH) as introduced previously in [7]. A subsumption of the DBH approach is as follows: All electron states in the energy range *E* = 0...*V* contribute to the apparent barrier height, φ_app_ = 1/α × (∂*_z_I*/*I*)^2^, weighted by their individual tunneling probability and the product of the DOS ρ_S_(*E*) × ρ_T_(*E*–*V*) [7]. Consequently, the apparent barrier height is relatively insensitive to changes of the DOS. The DBH, φ_diff_ = 1/α × (∂*_z_*∂*_V_I*/∂*_V_I*)^2^, however, selects predominantly contributions from the limiting energies *E* = 0 and *E* = *V* and, thus, is extremely sensitive to changes of the DOS at the Fermi levels. Figure 6 shows the DBH as calculated from measured ∂*_V_I*–*z* curves taken at the given biases (blue dashed curve) and the DBH as calculated from the deconvolved DOS shown in Figure 4 with the given values for β, γ, and *z*_0_ = 0.6 nm. Additionally, we calculated and plotted the DBH for constant DOS and the given separation (black dash-dotted curve) according to:

[8]
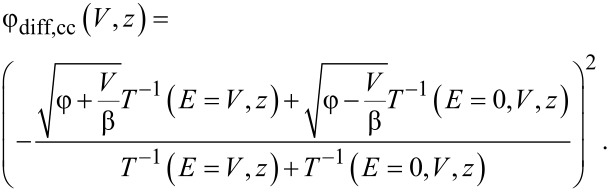


This entity can be interpreted as the square of a weighted average of the inverse tunneling decay lengths. In the case of varying DOS an additional weight factor would appear in front of the inverse decay lengths containing the DOS.

The agreement between the experimental and the calculated DBH from the deconvolved DOS is, however, unsatisfactory. This is a clear indication that the TPF used in the calculation does not yet reflect the true TPF accurately. The most prominent difference occurs in the energy range *E* > 0.5 eV where the experimental DBH falls off linearly as φ_diff_ ≈ 4.4 eV − 0.7*E*, whereas the calculated DBH increases linearly. Note that the DBH depends on the tip DOS, which decreases exponentially in the corresponding negative energy range (see Figure 4b). The exponential decrease of the tip DOS at negative bias favors increasingly deeper-lying states at the Fermi level of the sample and thus pushes the DBH towards the higher tip barrier. If this exponential decrease does not describe reality correctly, one has to compensate for the changing DOS by an appropriately changing tunneling probability. A first guess could be adjusting φ and γ such that they fit to φ_diff_ = φ + *V*/2 − γ*V* [20]. In our case we would obtain γ ≈ 1.2, which is too great since the weighting is neglected. Consequently, we took half of the change, giving γ ≈ 1.1 for our calculations at positive bias. However, it must be left to future work to find a better adjustment of the TPF, e.g., by using an energy dependent γ = γ(*E*), or to consider a possible dependence of the TPF on the symmetry of the involved electronic states [27].

## Conclusion

A formalism that was introduced in [13] to recover and deconvolve the DOS of tip and sample from *I–V* spectra was extended to allow application also to *z–V* spectroscopy data within the framework of the WKB approximation including the trapezoidal approximation. Successful recovery/deconvolution was demonstrated with simulated data. The corresponding results provide information on how the energy position of characteristic DOS features depends on relevant parameters such as the barrier height and width, the tip–sample separation, and true energetic position of such a feature. It is instructive to see here how the sensitivity of STS to variations of the DOS changes with energy and how little the occupied states contribute to the tunneling current and its derivative. The newly developed scheme was applied to experimental data obtained on Nb(110) by *z*–*V* spectroscopy and compared to corresponding results based on the previous formalism introduced for *I*–*V* spectroscopy. We find comparable accuracy for both approaches. Thus, the advantages of *z*–*V* spectroscopy as mentioned in the introduction can, if necessary, be exploited without losing accuracy in the course of the deconvolution procedure.

Comparison with earlier experimental [13] and theoretical results suggest, however, that the major deficiency of the STS analysis lies in the assumed transmission probability function (TPF). Thus, the problem has shifted from developing a deconvolution procedure to finding an adequate TPF that describes tunneling between tip and sample in a more realistic way. To tackle this problem it is presently suggested to elaborate on the properties of the differential barrier height (DBH) as obtained from measurements and the deconvolved DOS. For a proper deconvolution procedure, both DBHs should agree. In the case of discrepancies, however, one should re-iterate the procedure with an adjusted TPF until satisfactory agreement is obtained. Failure of such an adjustment could imply a principal limitation of the WKB approximation.

## References

[1] Binnig G, Rohrer H, Gerber C, Weibel E (1982). Phys Rev Lett.

[2] Geerk J, von Löhneysen H (2007). Phys Rev Lett.

[3] Tersoff J, Hamann D R (1985). Phys Rev B.

[4] Martensson P, Feenstra R M, Stroscio J A, Kaiser W J (1993). Methods of Experimental Physics.

[5] Feenstra R M (1994). Phys Rev B.

[6] Ukraintsev V A (1996). Phys Rev B.

[7] Koslowski B, Dietrich C, Tschetschetkin A, Ziemann P (2007). Phys Rev B.

[8] Wagner C, Franke R, Fritz T (2007). Phys Rev B.

[9] Passoni M, Bottani C E (2007). Phys Rev B.

[10] Passoni M, Donati F, Li Bassi A, Casari C S, Bottani C E (2009). Phys Rev B.

[11] Wahl P, Diekhöner L, Schneider M A, Kern K (2008). Rev Sci Instrum.

[12] Naydenov B, Boland J J (2010). Phys Rev B.

[13] Koslowski B, Pfeifer H, Ziemann P (2009). Phys Rev B.

[14] Doughert D B, Maksymovych P, Lee J, Yates J T (2006). Phys Rev Lett.

[15] Ploigt H-C, Brun C, Pivetta M, Patthey F, Schneider W-D (2007). Phys Rev B.

[16] Feng M, Zhao J, Petek H (2008). Science.

[17] Ziegler M, Néel N, Sperl A, Kröger J, Berndt R (2009). Phys Rev B.

[18] Simmons J G (1963). J Appl Phys.

[19] Simmons J G (1964). J Appl Phys.

[20] Koslowski B, Tschetschetkin A, Maurer N, Ziemann P (2011). Phys Chem Chem Phys.

[21] Koslowski B, Dietrich C, Ziemann P (2004). Surf Sci.

[22] Koslowski B, Dietrich C, Tschetschetkin A, Ziemann P (2006). Rev Sci Instrum.

[23] Kilimis D A, Lekka C E (2007). Mater Sci Eng, B.

[R24] 24Heinze, S. (unpublished), compare Reference [13].

[25] Lekka C E, Mehl M J, Bernstein N, Papaconstantopoulos D A (2003). Phys Rev B.

[26] Pan X, Johnson P D, Weinert M, Watson R E, Davenport J W, Fernando G W, Hulbert S L (1988). Phys Rev B.

[27] Donati F, Piccoli S, Bottani C E, Passoni M (2011). New J Phys.

